# Renal Organotherapy

**Published:** 1905-08-12

**Authors:** 


### RENAL ORGANOTHERAPY.

Since the discovery that the kidney habitually produces an internal secretion, a good many attempts have been made to mitigate the evils of renal insufficiency by the administration of renal tissues or extracts from them, but, on the whole, with little success. Dr. T. Choupin [1] has lately published the results of a prolonged research into the therapeutic value of the treatment, and speaks well of it. The lines of the procedure adopted by this author are those indicated by Professor Renaut in a communication to the Academy of Medicine in October 1903. One, two, or three kidneys are used for each day's supply, two being the usual number. Pigs' kidneys are used: they must be perfectly fresh, and should, if possible, be those of young animals. The kidneys are decapsulated, chopped, and washed in distilled water; they are then pounded in a mortar with 450 cc. of salt solution, whose strength is 7 parts per thousand. After being well pounded, the mixture is allowed to stand for four hours in a cool place and then decanted or filtered. The resulting extract is administered in three or four doses during the 24 hours, either alone, or in combination with some simple soup in cases where sentimental objections to the unmixed extract have to be combated. It is an essential of the treatment that the kidneys should be raw and fresh, and it is recommended that the extract be given for periods of ten days, followed by a five days' interval.

By this means Dr. Choupin claims to have effected very satisfactory progress in the treatment of chronic renal insufficiency. The paper contains detailed reports of ten cases, selected from the author's practice as good illustrations on account of the severity of the symptoms. Here is one: A man aged 57 underwent in 1898 a severe attack of influenza. Since that time he has been the subject of cardiac irregularity and dyspnœa on exertion, but without albuminuria, and without evidence of any valvular lesion. In January 1903 signs of heart-failure made their appearance: his liver became enlarged, œdema of the legs ensued, and the heart showed signs of dilatation. The urine became very albuminous, and the patient evinced a liability to attacks of urgent dyspnœa. From May 9th to May 30th he was treated by means of a careful dietary, rest, and the administration of all the ordinary drugs relied upon for such a condition, but without result. The albuminuria was progressive, and the quantity of urine fell to a litre a day in spite of a milk dietary. On May 30th treatment by kidney extract, prepared in the way above described, was instituted, and the experiment resulted in immediate improvement. The excretion of urine rose to 1,400 cc. in a very short time, and gradually mounted to 1,800 cc.; the albuminuria practically disappeared, and with it the dyspnœa. The administration of the kidney extract was continued for three months, and the health of the patient was reported to be good six months after the commencement of the renal therapy.

Without detailing the other cases upon which they are based, we may briefly outline the heads of the author's conclusions. These are, that extract of pigs' kidneys, prepared according to the method described, is one of the most active remedies for renal insufficiency so far proposed. It is a rapid diuretic, and, if given over a length of time, will generally restore the secretion of urine to its normal quantity, and keep it in its restored profusion for considerable periods of time. It exercises no harmful effects upon damaged kidneys. It will remove albumin from the urine of albuminuric subjects, and keep it free for a long interval, and by resting the damaged epithelium may be expected to assist in the recovery of the latter, so long as sufficient organic alteration to render recovery impossible has not already occurred. It is an antitoxin for the products of renal insufficiency, and its active principle is probably derived from the epithelium of the convoluted tubules. This antitoxin is not destroyed by the processes of digestion, and thus ingestion by the mouth offers an easy method of administration. It may give rise to minor disturbances—such as pruritus, urticaria, sweats, or gastritis—but the author knows of no instance in which anything in the way of a serious disturbance of health has followed the administration.

Whatever be the real merits of this treatment it has, at all events, the merit of being easy of application and apparently harmless, while in view of the inveterate character of the conditions for which it is recommended renal extract may well command an honest trial.

[1] Revue de Méd., Jan. and Feb. 1905.

